# Comment on: Cachexia index for prognostication in surgical patients with locally advanced oesophageal or gastric cancer: multicentre cohort study

**DOI:** 10.1093/bjs/znae136

**Published:** 2024-05-30

**Authors:** Kexun Li, Xuefeng Leng, Yongtao Han, Lin Peng

**Affiliations:** Department of Thoracic Surgery, Sichuan Clinical Research Centre for Cancer, Sichuan Cancer Hospital and Institute, Sichuan Cancer Centre, Affiliated Cancer Hospital of University of Electronic Science and Technology of China (Sichuan Cancer Hospital), Chengdu, China; Department of Thoracic Surgery, Sichuan Clinical Research Centre for Cancer, Sichuan Cancer Hospital and Institute, Sichuan Cancer Centre, Affiliated Cancer Hospital of University of Electronic Science and Technology of China (Sichuan Cancer Hospital), Chengdu, China; Department of Thoracic Surgery, Sichuan Clinical Research Centre for Cancer, Sichuan Cancer Hospital and Institute, Sichuan Cancer Centre, Affiliated Cancer Hospital of University of Electronic Science and Technology of China (Sichuan Cancer Hospital), Chengdu, China; Department of Thoracic Surgery, Sichuan Clinical Research Centre for Cancer, Sichuan Cancer Hospital and Institute, Sichuan Cancer Centre, Affiliated Cancer Hospital of University of Electronic Science and Technology of China (Sichuan Cancer Hospital), Chengdu, China

*Dear Editor*

We have read with great interest the article by Brown *et al.*^1^. Although they have conducted a meaningful study, there are certain points that require further discussion.

First, the study solely presented clinical staging data without including pathological staging. Although clinical staging is essential, pathological staging also offers substantial reliability and prognostic significance. Integrating both clinical and pathological staging could offer a more thorough evaluation of disease severity and prognosis in these patients. However, merging these stages might complicate the interpretation of surgical outcomes and prognostic evaluations.

Second, the disparities in baseline characteristics between the low- and normal-cachexia index groups, raise concerns regarding the potential impact of confounding variables on the study outcomes. Factors including age, Charlson Co-morbidity Index score, Eastern Cooperative Oncology Group performance status score, weight, histology, albumin levels, and neutrophil–lymphocyte ratio exhibited significant differences across the groups. Employing techniques such as propensity score matching or inverse probability of treatment weighting could help address these discrepancies and bolster the robustness of the study findings.

Finally, although the study extensively detailed the smoking status of patients, which is notably relevant to respiratory system tumours, the impact of alcohol consumption, more pertinent to digestive system tumours, was not discussed thoroughly. Further exploration and discussion of the potential influence of alcohol consumption on outcomes in patients with oesophageal or gastric cancer could provide additional insights and enrich the study findings.
